# Object Detection Based on Roadside LiDAR for Cooperative Driving Automation: A Review

**DOI:** 10.3390/s22239316

**Published:** 2022-11-30

**Authors:** Pengpeng Sun, Chenghao Sun, Runmin Wang, Xiangmo Zhao

**Affiliations:** School of Information Engineering, Chang’an University, Xi′an 710064, China

**Keywords:** object detection, roadside LiDAR, cooperative perception, a review

## Abstract

Light Detection and Ranging (LiDAR) technology has the advantages of high detection accuracy, a wide range of perception, and not being affected by light. The 3D LiDAR is placed at the commanding height of the traffic scene, the overall situation can be grasped from the perspective of top view, and the trajectory of each object in the traffic scene can be accurately perceived in real time, and then the object information can be distributed to the surrounding vehicles or other roadside LiDAR through advanced wireless communication equipment, which can significantly improve the local perception ability of an autonomous vehicle. This paper first describes the characteristics of roadside LiDAR and the challenges of object detection and then reviews in detail the current methods of object detection based on a single roadside LiDAR and multi-LiDAR cooperatives. Then, some studies for roadside LiDAR perception in adverse weather and datasets released in recent years are introduced. Finally, some current open challenges and future works for roadside LiDAR perception are discussed. To the best of our knowledge, this is the first work to systematically study roadside LiDAR perception methods and datasets. It has an important guiding role in further promoting the research of roadside LiDAR perception for practical applications.

## 1. Introduction

In recent years, the emerging technologies represented by autonomous driving have developed rapidly and become the key technologies to support the development of a new generation of intelligent transportation in the future. In China’s Energy Saving and New Energy Vehicle Technology Roadmap, it is expected that the market share of fully autonomous vehicles in China will reach 10% by 2030 [1]. In 2021, ERTRAC released the EU roadmap for autonomous driving, aiming to enter society by 2030 with autopilot as a standard [2]. Autonomous driving has become the inevitable trend of future automobile development. Object detection and motion prediction are the core of the perception system of autonomous vehicles, and real-time, accurate, and blind-spot-free object detection is the key to ensuring the safe driving of the autonomous vehicle. Currently, the maximum sensing distance of the onboard sensors is less than 150 m, which is easily affected by obstacles, adverse weather, and light, as well as the surface reflection intensity and motion state of the perceived object. Therefore, it is urgently necessary to develop vehicle–road collaboration technology to break through the technical bottleneck of insufficient awareness of the autonomous driving environment and improve the safety and reliability of autonomous vehicles [3]. The cooperative mode of intelligent cars and smart roads is the most effective means to ensure the safe operation of autonomous vehicles. The regional cooperative perception of intelligent roadside infrastructure makes up for the lack of local perception ability of a single vehicle and provides a new strategy to solve the over-the-horizon perception problem faced by autonomous vehicles [4].

At present, the intelligent roadside perception system mainly relies on traditional traffic information collectors, such as radar [5,6], cameras [7,8,9], and their fusion [10,11,12,13]. Radar can only capture moving objects in a certain area with low accuracy. The camera can obtain the color, brightness, texture, and other information of the real world, but it is easily affected by light and does not have good stability in extreme situations such as darkness and illumination mutation. Therefore, it has a natural disadvantage in the all-weather and high-precision perception of traffic information. LiDAR has the advantages of high detection accuracy, a wide sensing range, and no influence of light [14]. It has been widely used in object detection [15,16] and vehicle localization in autonomous vehicles [17,18], etc. When the 3D LiDAR is placed at the commanding point of the traffic scene, the overall situation can be viewed from the perspective of top view, and the high-precision motion trajectory of each object in the traffic scene can be sensed in real time. The information can be distributed to the surrounding vehicles or another roadside LiDAR through the advanced “5G + Cellular Vehicle-to-Everything (C-V2X)” wireless communication technology, to realize the vehicle–road cooperative regional perception. This can significantly improve the local sensing ability of autonomous driving. In addition, the roadside sensors are in a static state when working, so the perception accuracy and reliability of scene objects are higher [19,20].

Roadside LiDAR has unique characteristics compared with onboard or airborne LiDAR, such as the sparser distribution of the output point cloud and the lack of diversity of the background point cloud. The goal of deploying LiDAR sensors on the roadside for cooperative autonomous driving to achieve object detection and tracking is still in the exploratory stage, and there are still many difficulties for practical application. At present, there are some research reviews on object detection based on onboard LiDAR, as shown in [21,22,23], but there are few reviews on roadside LiDAR perception. Recently, Bait et al. [24] reviewed object detection and tracking based on roadside sensors. However, the detection methods and datasets related to roadside LiDAR are less involved. Therefore, this paper reviews the challenges, methods, and datasets involved in object detection using roadside LiDAR in detail, aiming to establish an overall landscape for object detection based on roadside LiDAR and accelerating the commercial application of roadside LiDAR-based perception technology. To the best of our knowledge, this is the first work to provide an in-depth survey of roadside LiDAR perception. The contribution of this paper is summarized as follows:

✧ The characteristics of roadside LiDAR, the challenges in object detection tasks, and methods of object detection based on roadside LiDAR in recent years are reviewed in depth, including the methods based on a single roadside LiDAR and cooperative detection of multiple LiDAR.
✧ The influence of adverse weather on LiDAR and methods of LiDAR perception in adverse weather are reviewed. Moreover, this paper collects and analyzes the currently published datasets related to roadside LiDAR perception.
✧ The existing problems, open challenges, and possible research directions of object detection based on roadside LiDAR are discussed in depth to serve as a reference and stimulate future works.

The remainder of this paper is organized as follows. Section 2 summarizes the characteristics of roadside LiDAR and the challenges of object detection based on roadside LiDAR. Section 3 reviews the object detection methods and datasets based on roadside LiDAR. Section 4 presents some open challenges and possible research directions for future works, and the last section concludes this paper.

## 2. Characteristics of Roadside LiDAR and Challenges in Object Detection Task

**Figure 1:**
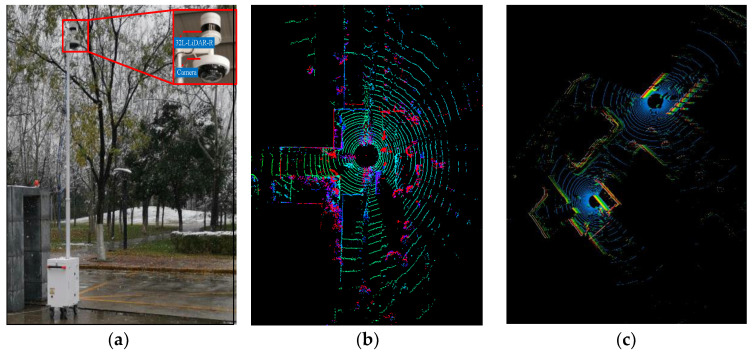
Roadside LiDAR and output point cloud. (**a**) 32L-LiDAR-R; (**b**) point clouds from a single LiDAR output; (**c**) point clouds from multiple LiDAR outputs.

As a perception sensor with high precision, high sensitivity, and a short delay, roadside LiDAR is generally deployed at urban intersections with a complex traffic flow, ramp entrances and exits of expressways, and accident-prone areas on expressways. Through the appropriate layout and networking of multiple roadside LiDARs, we can obtain the traffic data of the road section or the whole intersection from the perspective of top view without blind areas, and then reconstruct the scene of all objects and environments in the surrounding area, so as to accurately identify the vehicle status and vulnerable traffic groups such as pedestrians and non-motor vehicles in real time, as shown in Figure 1c. On the one hand, this can make up for the deficiency of perception of a single autonomous vehicle, and, at the same time, it can improve the level of road intelligence, effectively alleviate traffic congestion, and improve the capacity and efficiency of the road network. To meet the requirements of roadside perception, roadside LiDAR needs to work for a long time, even without interruption. In addition, due to the dynamic complexity of the traffic environment, the roadside LiDAR needs to have a larger sensing range and higher resolution. To cover a larger area, the roadside LiDAR is generally installed on the roadside infrastructure with a height of more than meters. Figure 1a shows a roadside 3D LiDAR with high-stability 32L-LiDAR-R, which was developed by China’s Wanji Technology for roadside perception applications in an intelligent vehicle–road cooperation system. It has 32 scanning beams, with a vertical field of view of 37° and a horizontal field of view of 360°. Its maximum coverage is 150 m. In addition, the difference between the roadside LiDAR and the onboard LiDAR is that the vertical scanning field of the roadside LiDAR is downward (−37~0°), and the laser transmitter adopts a multi-level distribution mode with different vertical angle resolutions (0.6°, 1°, 2°, 3°), which not only increases the density of traffic object points but also improves the spatial distribution of point clouds. The input point cloud is shown in Figure 1b.

At present, roadside LiDAR is mainly deployed on road sections of some pilot demonstration areas, and there are still many challenges in its deployment on a large scale for cooperative autonomous vehicles to achieve the goal of over-the-horizon perception in traffic scenes. This is manifested in the following:

(1) The best LiDAR sensor type for roadside perception remains an open question; except for a few LiDAR models especially developed for roadside sensing (e.g., 32L-LiDAR-R), most of the roadside collaborative projects directly deploy the onboard LiDAR at the roadside. LiDAR can be divided into mechanical rotary and solid-state LiDAR according to its structure. Mechanical rotary LiDAR changes faster and more accurately from “line” to “surface” by continuously rotating the transmitting head and arranging multiple beams of laser in the vertical direction to form multiple surfaces, to achieve the purpose of dynamic scanning and dynamic information reception. Commercially available rotary LiDARs include 16, 32, 40, 64, 80, and 128-beam LiDARs, which can perform 360-degree rotary scanning to achieve the three-dimensional reconstruction of full-scene traffic objects; the detailed parameters of the typical LiDAR are shown in Table 1. Hybrid solid-state LiDAR uses semiconductor “micro-motion” devices (such as a MEMS scanning mirror) to replace the macro-mechanical scanner and changes the emission angle of a single emitter through a micro-galvanometer, to achieve the effect of scanning without the external rotating structure. Therefore, this type of LiDAR can achieve a higher resolution and achieve image-level point cloud output. For example, the Falcon-K LiDAR has a higher resolution equivalent to 300 beams, a horizontal angle of view of 120 degrees, an angular resolution of 0.08 degrees, and an output of 3 million points per second in the double echo return mode.

**Figure 2:**
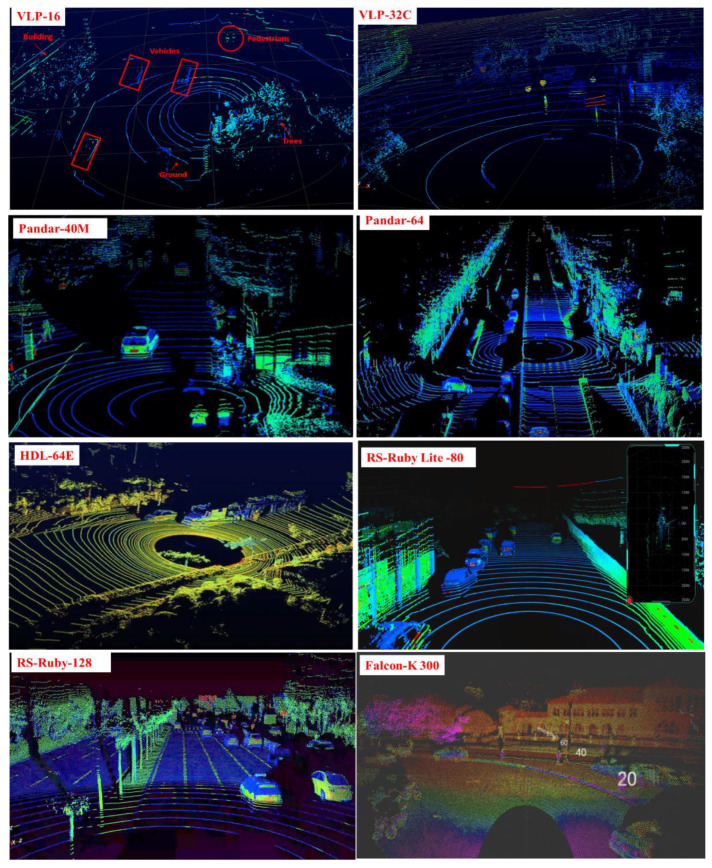
Point cloud of the typical LiDAR.

The number of LiDAR beams determines the resolution, detection range, and cost of the LiDAR. Figure 2 shows the output point cloud of a typical LiDAR. There is a large difference in the resolution and detection range of the object for LiDAR with different laser beams. The more laser beams, the higher the resolution, the greater the density of the laser point cloud, and the higher the cost. Because there is no uniform specification to specify which sensor will dominate future roadside sensing applications, the LiDAR sensing algorithms studied at present are all based on a certain beam of LiDAR, and the algorithms developed based on a certain type of LiDAR cannot be directly applied to another, different type of LiDAR. Therefore, it is still a great challenge to explore and develop a sensing method that can be applied to different types of LiDAR sensors.

(2) In order to allow the roadside LiDAR to obtain a sufficiently large coverage area, the deployment height of the roadside LiDAR is generally above 5 m. Therefore, compared with the point cloud output by onboard LiDAR, the point cloud is more widely distributed and sparser, which increases the difficulty of object detection. In addition, the roadside LiDAR is installed at a fixed location, resulting in a high degree of similarity and lack of diversity in the background point cloud. Moreover, the lack of large-scale roadside LiDAR point cloud datasets limits the use of deep learning methods, which causes the robustness and scene generalization ability to existing detection algorithms to still face enormous challenges in practical applications.
(3) Roadside LiDAR is fixed and deployed on the roadside, and it needs to work for a long time and in all weather. It will inevitably encounter adverse weather conditions such as rain, snow, and fog. Studies have shown that adverse weather has a greater impact on the performance of LiDAR. Rain, snow, fog, and other adverse weather will reduce the reflection intensity of the object point cloud and the number of object points, while increasing noise and reducing the resolution of the object in the point cloud, as shown in [31,32,33,34]. Therefore, the developed algorithm needs to maintain high reliability and accuracy in adverse weather.
(4) Most of the roadside LiDAR detection methods proposed at present are based on a single LiDAR, but the field of view of a single LiDAR is limited, and the point cloud data obtained by a single LiDAR have certain defects. The accuracy of perception can be significantly improved by fusing multiple LiDAR point clouds with the diversity of the surrounding space to achieve collaborative perception.

## 3. Object Detection Based on Roadside LiDAR

Three-dimensional object detection and motion prediction is a typical task of roadside LiDAR, which is used to obtain the precise location, three-dimensional size, category, and velocity of the object in the traffic scene. Object detection using roadside LiDAR can be divided into a single LiDAR-based method and multi-LiDAR cooperation-based methods. This section reviews the related challenges involved in the above tasks and the methods and datasets proposed in recent years in detail. In addition, this section also analyzes the impact of adverse weather on LiDAR and reviews the current work on LiDAR object detection in adverse weather.

### 3.1. Object Detection Methods Based on a Single Roadside LiDAR

**Figure 3:**
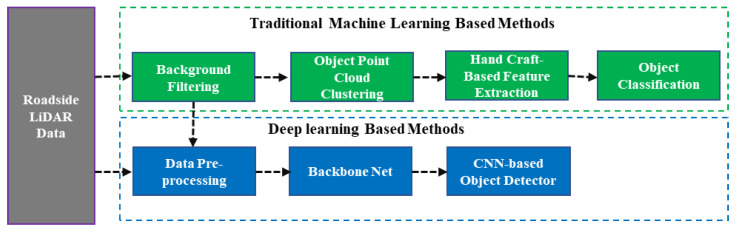
Technical route based on a single roadside LiDAR.

Compared with the regional sensing method based on multiple LiDAR cooperatives, the perception system using a single roadside LiDAR does not require the performance of complex spatio-temporal registration between multiple sensors and the requirements for deployment scenarios are much lower. Currently, most of the proposed object detection methods are based on a single roadside LiDAR, which can be divided into methods based on traditional machine learning and deep learning, and the technical route is shown in Figure 3.

A. Detection Method Based on Traditional Machine

In recent years, the research results of roadside LiDAR object detection methods based on traditional machine learning have been the most numerous. Usually, the object detection task is divided into four main steps: background filtering, object point clustering, feature extraction, and object classification [35,36,37,38]. When processing the point clouds captured by roadside LiDAR, some reasonable clusters are generated by background filtering and density-based spatial clustering methods, and then object detection is achieved by feature extraction and the classification of each cluster.

(1) Background filtering: The purpose of background filtering is to retain the interesting object points in the point cloud as much as possible while excluding other irrelevant points (buildings, trees, ground points), to improve the efficiency and accuracy of subsequent object segmentation. The principle of background filtering is mainly to model the background by using the characteristic that the spatial position of the background point in the time sequence point cloud changes little, and then judge whether the current point is the background point or the foreground point according to the difference in the depth, height, point density, and other characteristics between the current frame point cloud and the background model. According to the expression of the point cloud, background filtering methods can be divided into point cloud mapping-based methods and voxel-based methods.

**Figure 4:**
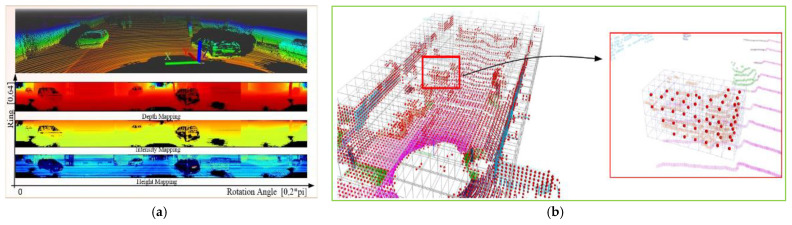
Representation of LiDAR point clouds. (**a**) Point cloud mapping. (**b**) Voxel-based [45].

The background filtering method based on point cloud mapping aims to encode the temporal point cloud according to the azimuth angle and the ID of the laser beam, as shown in Figure 4a. The background modeling and filtering are performed on the point cloud at the same beam and azimuth [39,40,41,42,43,44]. Zhao et al. [40] proposed an azimuth–height background filtering method. The main idea is to manually select the frame without a foreground point cloud as the background, and distinguish background points and foreground points by comparing the height of each point in the current point cloud with the background point in the background frame at the same beam and the same azimuth angle. Lee et al. [41] encoded the output point cloud according to the vertical angle and horizontal azimuth angle of the LiDAR, assumed that the background depth value at a given vertical angle and horizontal azimuth angle was constant, and took the median value of consecutive multi-frame sampling points at the same azimuth angle as the background depth value. Zhang et al. [43] used the same method to encode the multi-frame point cloud and construct the background model using the maximum distance according to the principle that the static environment is impenetrable. The above methods have high real-time performance, but, in urban scenes, it is difficult to ensure that only background points are obtained in the point cloud when the background frame is acquired. When the roadside LiDAR is deployed in crowded traffic scenes, object point clouds such as vehicles will be misjudged as background points, thus affecting the accuracy of the background model. Liu et al. [44] optimized the construction method of the background point cloud based on the maximum distance. By assuming that the vehicles and another object point only appear in the peer area, they introduced the filtering of the passing region to eliminate the object points introduced in the background point cloud. When the roadside LiDAR swings in the wind, the background model established by coding the LiDAR beams and azimuth angles may also be offset.

The background modeling method based on a three-dimensional voxel aims to divide the laser point cloud into three-dimensional voxels according to the spatial coordinates of the points, as shown in Figure 4b, and establish a background model based on density statistics using multiple point cloud frames, and then discriminate the background voxels and the foreground voxels according to the point density changes in each voxel [46,47,48,49]. In addition, Wu et al. [50] proposed a variable-dimension background filtering method, which uses a dynamic matrix to store the locations of background points, and identifies background points according to the number of neighboring points and the distance between the points in the current frame and the aggregation frames. This method uses a frame-by-frame update instead of multi-frame spatial aggregation to construct and update the background, which greatly reduces the amount of calculation for background point cloud extraction. Zheng et al. [37] used the background difference method in image processing to filter the background of the roadside LiDAR point cloud data. The above methods generally use multi-frame aggregation to filter background points by summarizing the farthest distance value, average value, point density, and other features. Because the threshold parameters mainly depend on the experience of engineers, most methods lack transferability. In addition, none of the above methods can handle scenes in which the foreground object is stationary for a period of time. Wang et al. [51] established a Gaussian background model with the average height and number of points as parameters for each three-dimensional voxel by considering the density and height distribution characteristics of the laser point cloud in three-dimensional space. These background modeling methods are based on a cubic grid. However, in the case of traffic congestion, it is difficult to choose the grid with the appropriate size to create the background model. Therefore, an adaptive polar grid Gaussian mixture model (APG-GMM) was proposed, which divides the three-dimensional grid based on the vertical angle and horizontal angle resolution of the roadside LiDAR, and a Gaussian mixture background model with the maximum distance value as the hyperparameter for the point cloud in each three-dimensional grid was established [52]. This method improves the accuracy of foreground and background segmentation. To further improve the accuracy of dynamic background modeling, Xia et al. [53] divided the points in the same scan line into multiple sectors and developed a new density background representation model (DBRM) to detect static and dynamic backgrounds (such as leaves) based on the assumption that the background objects are static in space and time. The static background is represented by density statistics, while the dynamic background is modeled by Gaussian mixture probability distribution.

Since LiDAR intensity is not as discriminative as the camera, most detection methods based on roadside LiDAR mainly use range information, while intensity values are often ignored. The intensity of laser radar is mainly affected by the surface reflectivity of obstacles, which is usually used for the detection of road signs, traffic signs, buildings, and other infrastructure, and is suitable for the background detection of roadside LiDAR. Zhang et al. [54] first encoded the 3D point cloud according to the LiDAR beam and azimuth angle where the point is located and used the hash function to store the depth value, intensity, and azimuth angle of the point in a beam–azimuth tensor with three channels. They decomposed the intensity channels based on the dynamic mode decomposition method to decompose the LiDAR data into the low-rank background and sparse foreground. Considering that the static infrastructure is the farthest object illuminated by LiDAR, the dynamic clustering method based on distance histogram analysis is used to separate the moving object from the static background. More details on the background modeling methods for roadside LiDAR are shown in Table 2. The accuracy of these methods varies from one scenario to another; thus, the accuracy is not listed in Table 2.

In summary, the above methods focus on the pre-modeling of the background point clouds, and it is difficult to achieve real-time background updates in changeable scenes.

(2) Object point cloud segmentation and recognition: After filtering the background point cloud, it is necessary to further identify vehicles, pedestrians, and other objects from the filtered foreground point cloud. Firstly, a three-dimensional object point cloud clustering algorithm, such as the point cloud clustering method based on Euler distance [55,56,57,58], point density, and its variants [36,43,44,59,60,61,62], is used to accurately segment the foreground object point cloud into independent objects. Then, according to the prior knowledge of the object, several handcrafted features, such as the standard deviation and clustering dimension of the cluster point cloud, are extracted from the cluster. Finally, some traditional classifiers, such as SVM, decision trees, and artificial neural networks, are used to realize object recognition. Zhang et al. [55,56] used the Euclidean distance method to cluster the object point cloud after filtering the roadside background point cloud based on the farthest point; extracted 28-dimensional features, such as the vertical distribution histogram, 3D size, and 2D minimum bounding box of the cluster points; and used the SVM classifier for vehicle detection. Upon finishing clustering based on Euclidean distance, Zhang et al. [54] estimated the bounding box to each cluster in which the total number of points is greater than the given threshold and used it as a candidate vehicle target for trajectory tracking. Table 3 shows a more detailed description of the proposed method.

Since the shape of point cloud objects is mostly irregular, the clustering method based on point cloud density can detect objects with arbitrary shapes, so it is also applied to the segmentation of roadside laser radar object point clouds. Among them, the Density-Based Spatial Clustering of Applications with Noise (DBSCAN) method is the most widely used [43,44,59,60,61,62].

Wu et al. [59,60] used the DBSCAN method to cluster and segment the roadside foreground point cloud and realized vehicle recognition based on six features: target length, height, distance from LiDAR, number of points, length–height ratio, and height profile of each cluster. Zhang et al. [43] used DBSCAN to cluster the roadside target point cloud; they adopted a different search radius according to the number of pedestrians and vehicles and filtered out the clusters belonging to noise points according to the total number of points after clustering. After DBSCAN clustering, Chen et al. [61] extracted features such as the center point of the cluster, the farthest distance between the cluster and the LiDAR center, the length and width of the minimum bounding box of the cluster, and the height difference, and classified the target based on SVM. Zhang et al. [62] also used DBSCAN to cluster the target point clouds. Five features were extracted and a probabilistic neural network (PNN) was used to achieve target classification. The performance of the DBSCAN algorithm depends on two parameters, the minimum number of points and the search radius, but the density of points decreases with the increase in the distance from the roadside LiDAR, which may affect the accuracy of density statistics, and the detection of distant vehicles and pedestrians becomes a major challenge. Therefore, Liu et al. [44] used the DBSCAN method to identify near-range traffic objects such as vehicles and road users. For far-range traffic object detection, they extracted the trajectory of the object according to the distance and moving direction, filtered out the noise by a fast Fourier transform (FFT), and identified the points of the object. Zhao et al. [36] improved the DBSCAN method for the roadside object point cloud segmentation task, divided the detection range into sub-regions based on the sensor distance, generated an ellipsoid search space with different radii in the vertical and horizontal directions, and estimated the point density according to the maximum number of points collected by the search ellipsoid, which improved the clustering accuracy of the target point cloud. In the target classification, the 2D distance, the total number of clusters, and the direction distribution characteristics of clusters are collected, and the backpropagation artificial neural network is used to realize the classification of pedestrians and vehicles. Both the Euclidean cluster and DBSCAN work directly in the 3D world, thus suffering from the large time costs of querying every point. To improve the clustering efficiency, researchers worked on the spherical range image representation of the LiDAR point cloud. This makes it convenient to borrow image processing techniques (e.g., region growing [63], connected-component labeling [64,65,66,67]) for faster clustering. Zhang et al. [63] coded the laser points filtered out of the background into a spherical range image according to the number of LiDAR beams and horizon angle resolution of the LiDAR and then used a counted region-growing method based on the distance threshold criterion to achieve object clustering. In [64,65], the angle formed by two adjacent laser beams in the range image is used as a criterion to separate the adjacent points belonging to different clusters. Both the distance threshold and the angle threshold are empirically based, and this empirical condition does not guarantee that it always works for all pairs of points. Therefore, Zhao et al. [67] proposed a divide-and-merge point cloud clustering algorithm, which first clusters the point cloud into many local components, and then merges the locally clustered components by voting on edge point pairs. The results show that the method outperforms all published methods on the SemanticKITTI [68] panoptic leaderboard. In addition, in view of the over-segmentation phenomenon in point cloud clustering, Li et al. [69] utilized connected-component labeling to quickly group points into initial clusters based on the distance difference and merge overlapping regions or neighboring parts based on the minimum mutual point Cartesian distance, thereby reducing the over-segmentation problem in the initial clusters. Shin et al. [70] combined a two-dimensional grid and undirected graph structure to cluster non-ground points first, and then used the Gaussian process regression method to merge the over-segmented parts to improve the segmentation accuracy of the object. The experimental results show that the method achieves real-time processing speed and high segmentation accuracy in most evaluation indicators.

The above research methods all use the traditional perception pipeline, which can produce stable results for the roadside LiDAR used in the research but suffer from generality. Some challenges and problems due to the handcrafted features may arise in practical applications when the LiDAR beam is changed. In addition, the threshold selection of background filtering and clustering is also subjective, feature selection depends on experience and professional skills, and the performance of the algorithm will decline in changing scenarios. These problems and challenges may limit the applicability of most of the above research methods in practical situations.

B. Detection method based on deep learning

Compared with the method based on traditional machine learning, the method based on deep learning can learn object features autonomously from a large number of data samples to achieve end-to-end object detection.

Deep learning methods have achieved remarkable results in the field of object detection based on vehicle-mounted LiDAR point clouds and images. On the one hand, the object detection method based on vehicle LiDAR is inspired by the image object detection method. Point clouds are mapped into a bird’s-eye view (such as BirdNet [71], BirdNet + [72], PIXOR [73], and YOLO3D [74]) or projected to a front view based on the horizontal and vertical angles of the points (such as LaserNet [75], FVNet [76], RangeDet [77]) to obtain a structured data representation, which is then fed into a feedforward convolutional neural network for 3D object detection. Although the projection-based method benefits from the mature 2D detector, it inevitably loses 3D spatial information due to the spatial quantization coding. To solve this problem, object detection methods based on voxels and LiDAR point clouds have been proposed. Representative networks include VoxelNet [78], PointPillars [79], Voxel-FPN [80], IPV-RCNN [81], etc. Since the resolution of the grid is much lower than the accuracy of the three-dimensional LiDAR point cloud, the accuracy of the object detection results is slightly reduced. The method based on the point cloud aims to directly process the LiDAR point cloud without any transformation and apply the multi-layer perceptron to directly extract the features of the points from the point cloud. Along this direction, many frameworks have been proposed for object detection [82] and semantic segmentation [83]. 

Although the deep learning method has achieved remarkable results in the processing of vehicle LiDAR point clouds, few studies have applied deep learning-based perception algorithms to roadside LiDAR systems. Because of the similarity and lack of diversity of the background point cloud output by the roadside LiDAR, it easily leads to the overfitting of the deep learning model. When the deployment location of the roadside LiDAR changes, the performance of the algorithm will be significantly reduced, which seriously limits the application of powerful deep learning methods in the roadside environment. To solve this issue, [51] first proposed to use the background filtering method to filter the background points, so as to increase the diversity of the roadside LiDAR point cloud. Then, the non-background point cloud is used as the input of the object detection network (e.g., PointPillars [79], SECOND [84], TANet [85]) for the training and testing of the model. Zhang et al. [57] adopted a similar approach for roadside LiDAR object detection and utilized PointVoxel-RCNN [86] (PV-RCNN) to detect vehicles and pedestrians from the extracted moving points. The experimental results show that the generalization ability of the above detection methods for target detection in different scenes has been improved. However, due to the lack of publicly available roadside LiDAR datasets for network training and testing, the detection accuracy of the algorithm is still lower than that of the vehicle LiDAR.

**Figure 5:**
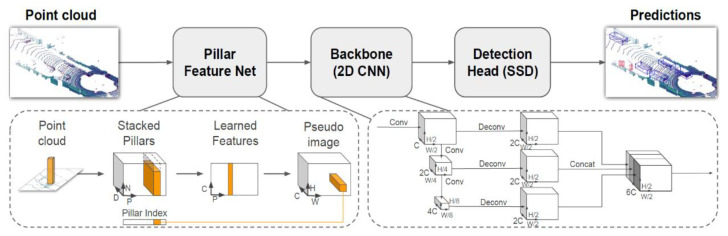
PointPillars 3D object detection model architecture [79].

Recently, Zhou et al. [87] explored and studied the training of a convolutional neural network (CNN) by utilizing a large-scale autonomous driving dataset and reusing it for vehicle detection from roadside LiDAR data. The proposed CNN model was modified from the PointPillars network [79], as shown in Figure 5, by adding dense connections to achieve more comprehensive feature extraction. It is worth noting that there are great differences between roadside LiDAR and onboard LiDAR in terms of installation height, output point cloud density distribution, data occlusion, and background point cloud. To empower the model with the capability of training on onboard datasets while inferencing on the roadside, Bai et al. [88] propose a Roadside Point-Cloud Encoder and Decoder (RPEaD) to transform roadside point clouds into a space in which the model trained on the onboard datasets can work. In order to reduce the shifting in Z-axis coordinates between the onboard LiDAR point cloud and the roadside LiDAR point cloud, the feature extraction network also adopts a structure similar to [79,87]; that is, the point cloud feature extraction network is based on pillars. Moreover, Zimmer et al. [89] designed a model, DASE-ProPillars, for 3D object detection using roadside LiDAR. They created a semi-synthetic infrastructure dataset with 6000 frames of the point cloud using the CARLA simulator, to make up for the deficiency of existing publicly available roadside LiDAR datasets. The comparison of the above methods is shown in Table 4.

### 3.2. Multi-LiDAR Cooperative Detection Method

**Figure 6:**
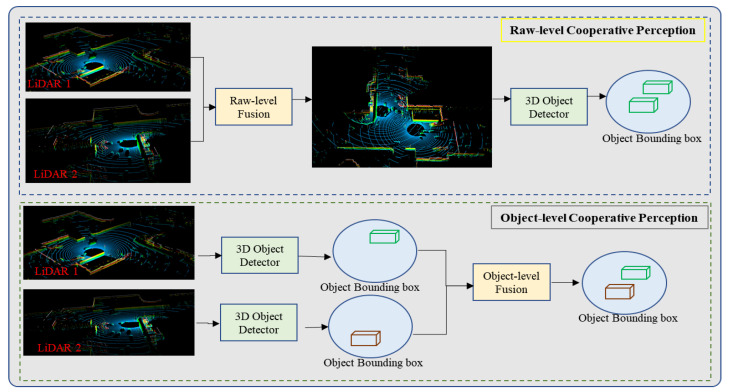
Logical illustration of raw-level and object-level schemes for cooperative object detection.

Due to the limited field of view of a single LiDAR, the acquired point cloud data have certain defects. The fusion of multiple LiDAR point clouds with spatial diversity characteristics to achieve regional cooperative perception can significantly improve the accuracy of scene perception. Cooperative perception can fuse data from multiple LiDAR sensors through V2I [90,91,92] or I2I [19,93,94], to realize information sharing and reduce sensor deployment costs. Based on the general procedure of 3D object detection, cooperative perception can be classified into three categories depending on the sensing information to be shared between LiDAR: raw, feature, and object-level cooperative perception [95]. Raw point cloud cooperative perception seeks to fuse LiDAR point clouds with different spatial distributions into point clouds with more comprehensive object information after alignment, and then apply the fused point clouds to object detection, as shown in Figure 6. Since the most abundant information of the same object is maintained after point cloud fusion, the object detection has the highest accuracy. However, a large amount of data needs to be exchanged between different sensors. The issue of how to realize real-time and distortion-free point cloud sharing under limited communication bandwidth is a huge challenge [96].

The object-level cooperative perception method first performs 3D object detection based on the point cloud output by each LiDAR to obtain information such as the position, bounding box, and confidence score of the object from different perspectives, and shares them to obtain more accurate object information [19,88,97], as shown in Figure 6. Since the scheme only needs to exchange the information of the object detected by LiDARs, the real-time cooperative perception among multiple LiDARs can be realized with a small communication bandwidth. However, when an object is missed in a laser radar due to occlusion of the perspective or low point density, it will be missed based on the object-level cooperative perception system. Therefore, the effect of cooperative perception based on the object level is worse than that based on point clouds and point cloud features [19].

**Figure 7:**
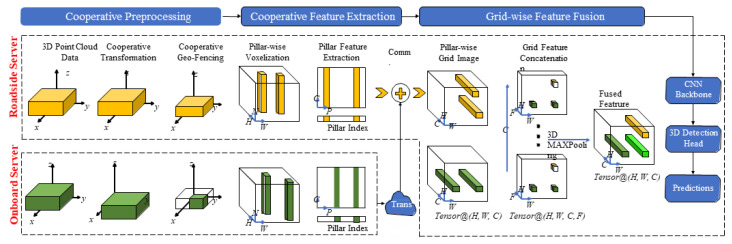
The system architecture of a feature-based cooperative perception framework [90].

To overcome the shortcomings of raw and object-level cooperative perception, the feature-level cooperative perception method needs to be further explored to achieve a trade-off between detection precision and bandwidth requirements. The feature-level cooperative perception first uses the feature coding layer based on voxel [98], pillar [90], or BEV [99] to generate feature maps from the point clouds output by each LiDAR, and then fuses them for object detection. Bai et al. [90] proposed a novel feature-level cooperative perception method from multiple 3D LiDARs. To avoid the extremely time-consuming 3D convolution, they first extracted the pillar features from the point cloud voxelized into pillars, and used a grid-wise feature fusion strategy to fuse multi-LiDAR data with high expandability; the architecture is shown in Figure 7. Chen et al. [98] extended their previous work [96] by fusing voxel features and using deep neural networks to learn deep features to achieve cooperative perception. Two sequence frames in the KITTI Vision Benchmark Suit (KITTI) dataset [100] were used to simulate the collaborative dataset to test the proposed method. However, KITTI only contains a limited number of traffic scenarios and is not specifically designed for cooperative perception. It is expensive to obtain a real dataset dedicated to multi-LiDAR cooperative perception, and it takes a great deal of time to manually label. Therefore, many methods proposed in recent years, such as [90,99,101], are based on synthetic data for more comprehensive cooperative perception experiments. Data generators and simulation tools such as CARLA [102] and SUMO [103] can not only generate a large amount of high-fidelity data for cooperative perception research under various traffic conditions but also provide accurate truth data. Marvasti et al. [99] used CARLA to generate simulated point cloud datasets to compare different data fusion strategies. The results show that both raw data ad deep feature fusion are significantly better than object-oriented fusion, especially when vehicle positioning errors are introduced. In addition, Wang et al. [104] also confirmed that, on the simulated dataset LiDARsim [101], the shared compressed depth feature map can achieve high-precision object detection while meeting the communication bandwidth requirements. However, due to the down-sampling of the raw point cloud data, the resolution of the shared feature map is low, so the strategy is not robust in high-precision object bounding box prediction.

In summary, deep feature fusion has achieved initial success, but there is still too much redundancy due to the sparsity of the shared feature map. These deep features are highly abstract and difficult to select, compress, and fuse through neural networks. For autonomous driving with real-time communication requirements, high-precision data sharing and low communication overhead are still huge challenges for cooperative perception technology.

### 3.3. Object Detection under Adverse Weather Conditions

**Figure 8:**
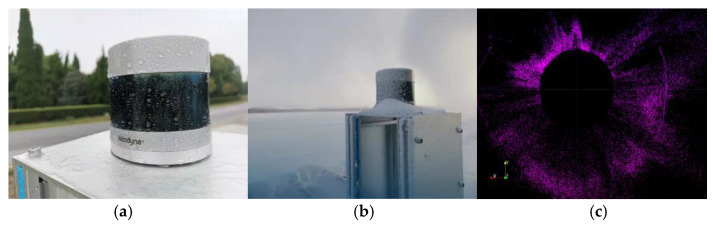
LiDAR operating in rain and snow and its output point cloud. (**a**) Under rain. (**b**) Under snow. (**c**) Point cloud under snow.

At present, the roadside LiDAR perception algorithm is mainly oriented toward normal weather conditions, while roadside LiDAR deployed on the road infrastructure will withstand various adverse weather types (such as rain, snow, fog, etc.). Nevertheless, the performance of LiDAR will be degraded to some extent in adverse weather, as shown in [25,31,32,33,34,105]. In foggy or snowy weather, the backscattering of light from water droplets or snowflakes received by LiDAR will produce a number of incorrect detection points, which also reduces the number of object points in the output point cloud and increases the difficulty of object detection, as shown in Figure 8. In addition, the effective detection distance and the reflection intensity of the object surface of the laser radar in adverse weather such as rain, fog, or snowfall will also be reduced. Therefore, to improve the target recognition accuracy of laser radar in adverse weather, the laser point cloud processing algorithm must deal with these influences.

In recent years, some work related to LiDAR point cloud enhancement in adverse weather has been carried out, mainly through the filtering algorithm to preprocess the LiDAR point cloud output to improve the accuracy of object detection in adverse weather. Park et al. [106] proposed a filter based on the point cloud reflection intensity, which uses point cloud reflection intensity characteristics to filter snow particles and retain important environmental characteristics based on the analysis of laser and snow particle characteristics. Robin et al. [107] proposed a CNN-based method to understand and filter noise in point clouds, and the results showed a significant improvement in performance compared with the latest geometry-based filtering. Roriz et al. [108] proposed a method of Dynamic Intensity Outlier Removal (DIOR), which combines the Dynamic Radius Outlier Removal (DROR) and Low-Intensity Outlier Removal (LIOR) algorithms to achieve higher accuracy while ensuring real-time performance. To improve the all-weather ability of roadside LiDAR, based on the analysis of the shortcomings of the existing filters, a combined denoising algorithm is proposed by combining the methods of crop box filter, ray ground filter, voxel filter, and statistical outlier filter [109]. Moreover, Wu et al. [110,111] studied the characteristics of roadside LiDAR data in rain and snow, proposed an improved density clustering method—3D-SDBSCAN—to distinguish vehicle points and snowflakes in LiDAR data, and used adaptive parameters to detect vehicles at different distances from roadside LiDAR sensors. The above methods are also mainly based on prior information and have poor adaptability to changeable scenes.

The rapid construction of an optimized detection network for large-scale LiDAR point cloud datasets in adverse weather provides a new idea for improving the performance of object detection in severe weather. Collecting and labeling sufficient training data in a diverse range of adverse weather conditions is laborious and prohibitively expensive. To address this issue, Yang et al. [112] modeled the performance of LiDAR under various fog conditions based on a 30-m artificial fog chamber established in Europe, and then loaded the pre-trained noise model on the LiDAR data recorded under clear weather conditions to quickly construct a large-scale LiDAR point cloud dataset under fog conditions. Kilic et al. [113] proposed a physics-based approach to simulate the LiDAR point clouds of scenarios in adverse weather conditions on existing datasets collected under normal weather conditions. Through the method, the accuracy and reliability of the 3D object detector can be improved by using the abundant existing real datasets collected in clear weather. Recently, Hahner et al. [114,115] proposed a physical method dedicated to fog and snowfall simulation, applicable to any LiDAR dataset. These partially synthetic data can be used to improve the robustness of the LiDAR point cloud perception algorithm in real fog and snowfall environments.

To summarize, there is a lack of large-scale roadside LiDAR point cloud datasets in poor weather. At present, the main method to improve the detection performance of LiDAR in adverse weather focuses on point cloud denoising. Although the roadside LiDAR point cloud collected in normal weather can be transformed to adverse weather through the physical modeling of LiDAR, there is still a lack of quantitative performance parameters of LiDAR in adverse weather such as rain and snow, which leads to a lack of confidence in the established LiDAR model. It is impossible to rely on the existing model to generate high-fidelity roadside LiDAR point clouds in adverse weather.

### 3.4. Datasets

**Figure 9:**
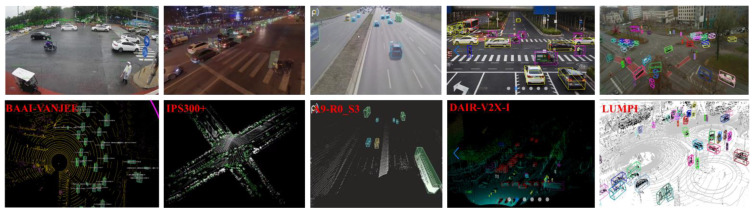
Dataset collected by roadside LiDAR and cameras.

High-quality roadside datasets have significant industrial value, which can accelerate the iterative optimization of roadside perception models in vehicle–infrastructure collaboration and play a positive role in promoting innovative research in academia. In recent years, many object detection and tracking datasets collected by LiDAR and cameras have been published, such as KITTI [100], H3D [116], ApolloScape [117], Waymo [118], NuScenes [119], PandaSet [120], and Panoptic Nuscenes [121]. However, these are based on data from onboard sensors; there are relatively few public roadside datasets, especially 3D point cloud datasets, which cannot meet the current needs for the iterative optimization of roadside perception models. Since last year, some datasets collected by roadside LiDAR and cameras have been released one after another. We have summarized the released datasets, and the data samples are shown in Figure 9.

**BAAI-VANJEE:** In 2021, Deng et al. [122] released a roadside perception dataset, called the BAAI-VANJEE dataset, to support the Connected Automated Vehicle Highway technologies. This dataset provides 2500 frames of point cloud data and 5000 frames of RGB images collected from a complex urban intersection and highway scenes in China, covering different weather conditions (sun, cloud, rain) and times (day, night). It was collected by a 32-beam roadside LiDAR sensor and two cameras placed on the roadside at approximately 4.5 m. More detailed parameters are shown in Table 5.

**IPS300+:** In order to promote the research on roadside multi-modal perception in cooperative vehicle infrastructure systems, Wang et al. [28] published a bimodal dataset in 2022 with a band of roadside LiDAR and cameras. The collection scene is an urban intersection covering an area of 3000 square meters, covering a radius of 300 m. Two perception units (IPU) are installed on the diagonal of the intersection at a distance of 5.5 m from the ground for data collection. Each perception unit consists of an 80-beam RoboSense Ruby-Lite LiDAR and two Sensing-SG5 color cameras. The proposed dataset includes 14,198 frames of data covering different times. The point clouds registered by the two IPUs are stored as a single PCD file in one frame for annotation, and each frame has an average of 319.84 tags, including seven categories of pedestrians, cyclists, tricycles, cars, buses, trucks, and engineering vehicles.

**DAIR-V2X-I:** To accelerate computer vision research and innovation for vehicle–infrastructure cooperative autonomous driving, Yu et al. [30] released the DAIR-V2X dataset in 2022. The dataset was collected from 10 km of urban roads, 10 km of expressways, and 28 intersections in Beijing’s high-level automatic driving demonstration area. Four pairs of 300-beam roadside LiDAR and high-resolution cameras were deployed at each of the 28 intersections. DAIR-V2X-I is a subset of DAIR-V2X, which is dedicated to roadside cooperative perception, and contains 10,084 frames of jointly annotated images and roadside LiDAR point cloud data, respectively. The annotator exhaustively labels each of the 10 object classes in each image and point cloud frame, including different vehicles, pedestrians, and different cyclists.

**A9 Dataset:** In 2022, Christian et al. [123] presented the A9 dataset based on roadside sensors from the 3-km-long Providentia++ test field near Munich in Germany. Sensors include a camera, radar, and 64-beam Ouster LiDAR, and are mounted on the gantry bridges and masts and provide vistas of the road. The dataset offers labeled images and LiDAR point clouds of multiple road segments and from different angle recordings of dense traffic on the A9 autobahn during daylight. The release R0 consists of 1098 labeled frames and 14,459 labeled 3D objects, including nine categories of objects, such as car, trailer, truck, van, pedestrian, bus, motorcycle, bicycle, and others.

LUMPI: Recently, Bush et al. [27] published a multi-view dataset; they used three different configurations of cameras and LiDAR to collect data, and all LiDARs were synchronized by GPS, covering the intersection area through different combinations of LiDAR and installation methods. They collected 145 min of data over three different days with varying weather conditions and labeled six categories of objects, including person, car, bicycle, motorcycle, bus, and truck. The detailed information is shown in Table 5.

## 4. Discussion and Future Works

Although the research on traffic object detection based on roadside LiDAR is still in the exploratory stage, it plays an increasingly important role in the realization of non-blind areas and the over-the-horizon perception ability of cooperative automatic driving vehicles. At present, many solutions have been put forward for this problem, but there are still many difficulties for commercial applications. This section will discuss in detail some very important but rarely studied open problems in object detection based on roadside LiDAR, as well as future work directions.

**(1)**  **Towards Scene-Adaptive High-Precision Perception:** Due to the homogeneity and lack of diversity of the background point clouds output by the roadside LiDAR, the existing detection methods mainly use traditional background filtering, clustering segmentation, feature extraction, and classification methods. This cascade method is easily affected by any changes and errors in the upstream model. The reliability and accuracy of the algorithm are greatly affected by the LiDAR deployment environment. These problems will limit the application of traditional perception methods in real scenes. Therefore, it is necessary to use the powerful learning ability of deep learning to develop an integrated object detection network with scene adaptability to improve the accuracy and reliability of object detection based on roadside LiDAR. Therefore, on the one hand, we can try to collect and annotate a large number of roadside LiDAR data from the simulation environment or the real scene to promote the research of roadside LiDAR perception methods based on data-driven considerations. On the other hand, based on the existing small number of labeled roadside point cloud datasets, we can attempt to carry out research on the deep learning method of roadside LiDAR based on few-shot learning [124].
**(2)**  **Adaptation in Different Roadside LiDARs:** According to the research review in this paper, the roadside LiDAR used in the existing methods or published datasets has great differences in the scanning mode and the number of scanning lines; specifically, the number of scanning lines of LiDAR ranges from 16 to 300. The observation angle of the object, sparsity of the output point cloud, and regional occlusion are different for the roadside LiDAR with a different number of scanning lines and different installation height, which makes it difficult for the existing perception methods to be applied to different roadside LiDARs. Thus far, there is no uniform standard specification for which type of LiDAR will dominate the roadside perception application in the future. Therefore, it is very important to promote research on the common perception algorithm of different roadside LiDARs, which can mine the characteristics of LiDAR point cloud data and try to build a domain-invariant data representation, so that the detection model trained based on existing LiDAR data can be reused for new LiDARs [125].
**(3)**  **Adaptation in Different Weather Scenarios:** The existing perception algorithms based on roadside LiDAR are usually developed for scenes under normal weather, and do not perform well in adverse weather conditions such as rain and snow. However, the construction of roadside LiDAR point cloud datasets under adverse weather is time-consuming and costly. Therefore, we can try to establish a physical model of LiDAR with high confidence under adverse weather based on the study of the impact of adverse weather on LiDAR parameters, and then use the existing roadside LiDAR point cloud collected under normal weather to quickly construct a large-scale LiDAR point cloud dataset under adverse weather. This will promote the research of roadside LiDAR perception algorithms for weather domain adaptation.
**(4)**  **Towards Multiple Roadside LiDAR Cooperation:** Through a review of the roadside LiDAR perception approach in recent years, most of the studies above are based on a single LiDAR sensor, while few of them use multiple roadside LiDARs to enhance perception. However, the field of view of a single laser radar is limited, and the point cloud data obtained have some defects. The accuracy of scene perception can be significantly improved by integrating multiple laser radar point clouds from different perspectives in the surrounding space to achieve cooperative perception. At present, high-precision data sharing and low communication overhead are major challenges for multi-side LiDAR cooperative perception. If we can explore the fusion strategy of object information and key point features based on the output of multiple LiDARs, and construct the object detection model of multiple roadside LiDARs based on the fusion of key point depth features, it will provide a new idea for the realization of the enhanced perception of traffic objects under low communication bandwidth.
**(5)**  **Towards Multi-Model Cooperation:** The perception system based on multi-modal sensor information fusion can significantly improve the perceived performance of a single modal sensor through the complementarity of different types of modal information (such as LiDAR point clouds and images) and appropriate fusion technology [126]. However, the performance of multi-modal fusion has been limited due to the spatiotemporal asynchrony between sensors, domain bias, and noise in different modal data. Future work can explore more effective spatiotemporal registration and data fusion strategies for different modal sensors, and thus achieve better perception performance.

## 5. Conclusions

This paper reviewed several current issues and trends of object detection based on roadside LiDAR. This review mainly includes the following parts: the characteristics of roadside LiDAR and the challenges of object detection, object detection based on a single roadside LiDAR, object detection based on multiple roadside LiDARs, the challenges of roadside LiDAR in adverse weather and related work, the roadside LiDAR dataset, and some open problems and future work directions. The major findings in the above studies are as follows.

(1) Due to the particularity of the deployment location of roadside LiDAR, most of the current object detection methods based on roadside LiDAR mainly adopt the traditional point cloud processing method, which has low accuracy compared with the existing object detection method based on onboard LiDAR and has poor adaptability to changing scenes and LiDAR with different beams.
(2) The roadside LiDAR is deployed on the roadside infrastructure for a long time, and the developed algorithm must consider the impact of adverse weather on the LiDAR. At present, there is a lack of roadside LiDAR point cloud datasets in adverse weather, and the work of roadside LiDAR detection in adverse weather is mainly focused on point cloud denoising.
(3) Most of the algorithms are based on the data of a single roadside LiDAR, and the cooperativity of the LiDAR can better exploit the advantages of the roadside LiDAR, so it is necessary to continue to promote research on the cooperative perception of multiple roadside LiDAR.
(4) Since last year, some roadside LiDAR and image datasets have been released, but the coverage of the scene is limited, the data collected in adverse weather are sparse, and the number of LiDAR lines used in each dataset is also very different.

Roadside LiDAR will play an important role in the future cooperative perception system. There are still many challenges in the practical application of the existing perception methods based on roadside LiDAR. It is hoped that the review in this paper will play a guiding role in further promoting the research of roadside LiDAR perception methods.

## Figures and Tables

**Table 1:** Overview of typical LiDAR sensors.

LiDAR	LiDAR Beams	FOV (Vertical)	FOV (Horizontal)	Resolution (Vertical)	Resolution (Horizontal)	Range	Range Accuracy	Points Per Second
VLP-16 [25]	16	30° (−15~+15)	360°	2°	0.1~0.4°	100 m	±3 cm	300,000
VLP-32C [26]	32	40° (−25~15)	360°	≥0.03°	0.1~0.4°	200 m	±3 cm	Single echo mode: 600,000
Pandar40M [26]	40	40° (−25~15)	360°	0.33~6°	0.2°	120 m	±2 cm	Single echo mode: 720,000
Pandar64 [27]	64	40° (−25~15°)	360°	0.167~6°	0.2°	200 m	±2/5 cm	Single echo mode: 1,152,000
HDL-64E [27]	64	26.8° (24.8~2°)	360°	0.33~6°	0.08~0.35°	120 m	±2 cm	Single echo mode: 1,300,000
RS-Ruby Lite [28]	80	40° (−25~15°)	360°	Up to 0.1°	0.1~0.4°	230 m	±3 cm	Single echo mode: 1,440,000
RS-Ruby [29]	128	40° (−25~15°)	360°	Up to 0.1°	0.2~0.4°	200 m	±3 cm	Single echo mode: 2,304,000
Falcon-K [30]	Eq. to 300	25°	120°	0.16°	0.24°	250 m	±2 cm	Double echo mode: 3,000,000

**Table 2:** Summary of background modeling for roadside LiDAR.

References	Data Description	Initial Frames	Criteria of Background/Foreground	Merit	Limitation
Zhao et al. [40]	Azimuth–height table	Manually select the frame without foreground points	Height information of the background frame	High real-time performance.	Manually select the initial frame and poor environmental adaptability.
Lee et al. [41]	Range image	Multi-frame point cloud	The median value and the average height of the sampling points in the accumulated frame.	High real-time performance, low complexity.	Ineffective background modeling for scenes with more dynamic targets in consecutive frames.
Zhang et al. [43]	Spherical range image	954-point cloud frames with few vehicle points	The maximum distance and mean distance of the sampling points in the accumulated frames.	High real-time performance, low complexity.	Not applicable to congested traffic scenarios.
Liu et al. [44]	Spherical range image	315-point cloud frames	The maximum range value of consecutive frames after filtering the object points.	Background modeling uses fewer point cloud frames and can adapt to different levels of traffic scenarios.	Decrease in background modeling accuracy when LiDAR swings due to wind.
Wu et al. [46,47,48,49]	Voxel/3D cube	2500-point cloud frames	The density of each cube is learned from accumulated frames.	High background modeling accuracy in areas with high point density at close range.	The size of the cube largely influences accuracy and computational cost.
Wu et al. [50]	Dynamic matrix	Randomly select a frame	The number of neighbors, and the distance between the points in the current frame and the aggregated frames.	Effectively filter the background points under different scenarios.	The value parameter mainly depends on experience and lacks portability.
Wang et.al [51]	Voxel/cubes	Multiple point cloud frames from different periods	Established a Gaussian background model with average height and number of points as parameters.	The robustness of the algorithm is good, and it still achieves good performance in the case of LiDAR shaking.	Different voxel sizes need to be set for different scenes, and it is difficult to select the appropriate size.
Wang et al. [52]	An adaptive grid	One frame of the LiDAR data	Established a Gaussian mixture background model with the maximum distance as the hyper-parameter.	No manual selection of background frames and high background extraction accuracy for sparse point clouds.	The algorithm has high time complexity.
Zhang et al. [54]	Elevation azimuth matrix	Successive frames	Established a background model by regressing the intensity features of continuous frames based on the DMD algorithm.	The algorithm process is applied directly on the scattered and discrete point clouds and has strong robustness.	Reflection intensity is attenuated in adverse weather, resulting in reduced algorithm performance.

**Table 3:** Summary of the features used for LiDAR data classification.

References	Object Clustering	Selected Features	Classifier	Applicability
Zhang et al. [55,56]	Euclidean cluster	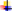 20D vertical point distribution histogram of the cluster. 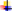 The standard deviation of points in X, Y, Z. 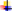 Volume size of the cluster: length, width, maximum height, and minimum height. 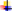 Area of the 2D minimum bounding box of the cluster.	SVM classifier with RBF kernel	Vehicle detection
Zhang et al. [54]	Distance-based	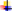 3D bounding box to each cluster. 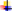 The number of points.	-	Vehicle detection
Wu et al. [59,60]	DBSCAN	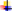 Object length, height. 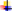 Difference between height and length. 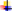 10D object height profile. 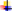 Distance between object and the LiDAR. 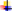 The number of points.	Naïve Bayes, K-nearest neighbor classification, decision tree, and SVM	Vehicle classification
Zhang et al. [43]	DBSCAN	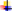 Compare the distances between objects and LiDAR and the distances between ground and LiDAR.	-	Vehicle and pedestrian detection
Chen et al. [61]	DBSCAN	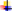 Average X, Y, Z of the points. 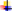 The nearest distance between the points and the LiDAR. 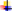 Object length and width. 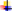 Difference between the height of the object and length.	SVM classifier	Vehicle detection
Zhang et al. [62]	DBSCAN	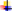 The number of points. 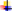 Maximum intensity change. 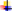 Distance between tracking point and LiDAR. 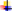 Maximum distance in the XY plane and maximum distance in Z-axis	Probabilistic neural network (PNN)	Pedestrian, bicycle, passenger car, and track classification
Zhao et al. [30,36]	Improved DBSCAN	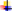 The number of points. 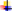 2D distance. 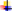 The direction of the clustered points’ distribution.	Backpropagation artificial neural network (BP-ANN)	Pedestrian and vehicle classification

**Table 4:** Comparison of object detection methods for roadside LiDAR based on deep learning.

References	Year	Architecture	Dataset	Strategy
Wang et al. [51]	2021	Background filtering module + 3D CNN detectors	Training: collected using roadside LiDAR (800 frames). Testing: collected using roadside LiDAR (100 frames).	● Filtering out the background points before training model to improve the generalization ability and performance of 3D detectors.
Zhang et al. [57]	2022	Background filtering, clustering, tracker module + PointVoxel-RCNN detector	Training: collected using a RS-LiDAR-32 roadside LiDAR (700 frames). Testing: collected using a RS-LiDAR-32 roadside LiDAR (63 frames).	● Candidate objects are generated by background filtering, moving point clustering, and UKF tracker before detection.
Zhou et al. [87]	2022	Modified PointPillars	Training: a large-scale autonomous driving LiDAR dataset, PandaSet, captured through a Panda 64 LiDAR (11200 frames). Testing: Roadside LiDAR data collected using a Velodyne VLP-32C. (1000 frames).	● Reusing CNNs pre-trained on autonomous driving data to detect vehicles from roadside LiDAR data. ● Dense connections between convolutional layers are introduced on the basis of PointPillar to achieve more effective features.
Bai et al. [88]	2022	RPEaD + PointPillars + FPN	Training: large-scale autonomous driving LiDAR dataset, Nuscenes. Testing: 130 frames manually labelled based on the drone’s view.	● Transform roadside point clouds into coordinates suitable for training on the onboard dataset by RPEaD. ● To further reduce the sensitivity of the model to the difference point clouds in height, they voxelized the point cloud to generate point cloud pillars. ● Designed feature pyramid network to generate predicted bounding boxes.
Zimmer et al. [89]	2022	DASE-ProPillars, an improved version of the PointPillars model	Training: a semi-synthetic dataset with 6000 frames generated by a OSI-64 simulated LiDAR sensor. A9 dataset, Regensburg. Testing: A9 dataset, Regensburg Next Project.	● Introduce five extensions to improve PointPillars. ● Create a semi-synthetic roadside LiDAR dataset to train the proposed model. ● 50 LiDAR frames collected from roadside LiDARs were manually labeled to fine-tune the detector. ● Perform transfer learning from the A9 dataset to the dataset from the Regensburg Next project.

**Table 5:** Survey of datasets recorded by infrastructure sensors.

Dataset	Year	LiDAR	Cameras	Annotated LiDAR Frames	3D Boxes	2D Boxes	Classes	Traffic Scenario	Weather and Times	Sensor Height
BAAI-VANJEE [122]	2021	1 32L-LiDAR-R 32-beam LiDAR	2 RGB cameras	2500 frames	74 k	105 k	12	Urban	Sunny/cloudy/rainy, day/night	4.5 m
IPS300+ [28]	2022	1 Robosense Ruby-Lite 80-beam LiDAR	2 color cameras	14,198 frames	454 M	-	7	Urban	Day/night	5.5 m
DAIR-X2X_I [30]	2022	1 300-beam LiDAR	1 RGB camera	10,084 frames	493 k	-	10	Urban highway	Sunny/rainy/fogy, day/night	-
A9-Dataset [123]	2022	1 Ouster-OS1 64-beam LIDAR	1 RGB camera	1098 frames	14 k	-	7	Autobahn highway	daylight	7 m
LUMPI [27]	2022	1 VLP-16, 1 HDL-64, 1 Pandar64, 1 PandarQT	1 PiCam 1ATOM 1YiCam	145 min	-	-	6	Urban	Sunny/cloudy/hazy/	-

## Data Availability

Not applicable.
